# Intraoperative desaturation in pediatric patients at high altitude: incidence, risk factors, and a non-linear body weight safety threshold

**DOI:** 10.3389/fped.2026.1871256

**Published:** 2026-06-23

**Authors:** Dawa Puzhen, Yiwen Yang, Xiaoyan Li, Laba Ciren, Yi Feng, Qi Yan

**Affiliations:** 1 Department of Anesthesiology, People’s Hospital of Xizang Autonomous Region, Lhasa, China; 2 Department of Anesthesiology, Peking University People’s Hospital, Beijing, China

**Keywords:** hemoglobin, high altitude, intraoperative desaturation, mechanical ventilation, pediatric anesthesia, Tibetan children

## Abstract

**Background:**

Pediatric patients undergoing general anesthesia are vulnerable to intraoperative hypoxemia, and this risk may be magnified at high altitude. However, the specific incidence, risk factors, and dose-response relationships for desaturation in children residing at extreme altitudes (≥3,650 m) remain poorly defined.

**Methods:**

This retrospective cohort study included 1,793 consecutive pediatric patients (<12 years) of native Tibetan ethnicity who underwent elective non-cardiothoracic surgery under general anesthesia with mechanical ventilation at an altitude of 3,650 m (Lhasa, China) between January 2020 and October 2025. Intraoperative desaturation was defined as SpO_2_ <90% lasting ≥1 min during mechanical ventilation. Multivariable logistic regression, restricted cubic splines, and piecewise regression were used to identify risk factors and nonlinear thresholds.

**Results:**

The overall incidence of intraoperative desaturation was 3.5% (63/1,793). Multivariable analysis revealed that younger age (adjusted OR per one-year increase 0.80, 95% CI: 0.72–0.90), severe underweight (<3rd percentile for age and sex; adjusted OR 4.57, 95% CI: 2.18–9.58), lower preoperative hemoglobin (adjusted OR per 1 g/dL increase 0.85, 95% CI: 0.74–0.98), prolonged mechanical ventilation (adjusted OR per 1 h increase 1.46, 95% CI: 1.24–1.73), and female sex (adjusted OR 1.76, 95% CI: 1.01–3.04) were independent risk factors. A non-linear L-shaped relationship was identified between absolute body weight and desaturation risk (*P* for non-linearity = 0.004), with an exploratory non-linear safety threshold at 26.95 kg. Below this threshold, each 1 kg increase in weight reduced the risk by 25.6% (adjusted OR 0.744, 95% CI: 0.646–0.857). In unadjusted between-group comparisons, desaturation was associated with higher rates of delayed extubation, a nearly four-fold higher ICU admission rate, and prolonged hospital stay (all *P* < 0.01).

**Conclusion:**

At extreme high altitude, intraoperative desaturation in pediatric patients is independently driven by younger age, severe underweight, lower hemoglobin, female sex, and prolonged ventilation. The identified L-shaped relationship and the exploratory 26.95 kg inflection point provide growth-based criteria for preoperative risk stratification. Together with the other independent risk factors (younger age, lower hemoglobin, female sex, and prolonged ventilation), these findings underscore the need for individualized, comprehensive perioperative strategies to reduce desaturation and improve outcomes in high-altitude pediatric anesthesia.

## 1 Introduction

Maintaining adequate oxygenation is a critical challenge in pediatric perioperative care (1). Even at sea level, the reported incidence of intraoperative hypoxemia during general anesthesia in pediatric patients ranges from 3.1%–12% (2, 3), which is closely linked to increased postoperative morbidity and healthcare burden (4, 5).

This vulnerability is substantially magnified in high-altitude environments. At an altitude of 3,650 m in Lhasa, Tibet, the inspired partial pressure of oxygen (PO_2_) is reduced. Native high-altitude residents exhibit physiological adaptations, including tachypnea, increased pulmonary diffusing capacity, and compensatory erythrocytosis (6). Nevertheless, the safety margin during general anesthesia remains critically narrow. Anesthesia uniformly decreases functional residual capacity (FRC) and promotes absorption atelectasis. In the setting of hypobaric hypoxia, these changes are further exacerbated by a diminished baseline oxygen reserve.

Despite these physiological risks, a significant knowledge gap persists. Extensive studies in sea-level populations have established that physical growth indicators, particularly body weight, are critical determinants of intraoperative hypoxemia (4, 7). This is of particular clinical concern given that chronic hypobaric hypoxia at extreme altitudes has been shown to profoundly impact child growth, resulting in a significantly higher prevalence of developmental restriction and lower body weight percentiles among native Tibetan pediatric populations (8). However, despite this compounded baseline vulnerability, it remains uncharacterized how these crucial developmental metrics influence respiratory vulnerability during mechanical ventilation at high altitudes (≥3,650 m).

Therefore, this study aimed to investigate the incidence, independent risk factors, and postoperative outcomes of intraoperative desaturation during mechanical ventilation in pediatric patients at 3,650 m altitude, with a focus on physical development indicators. Additionally, we sought to explore the specific dose-response relationship between body weight and the risk of desaturation to establish an explicit clinical safety threshold. To our knowledge, this is the first large-scale study to characterize intraoperative desaturation in this unique population. By identifying specific, modifiable risk factors and physiological breakpoints, we aim to provide evidence-based targets for preoperative risk stratification and targeted optimization in high-altitude pediatric anesthesia.

## 2 Materials and method

### 2.1 Study design

This retrospective study received approval from the Institutional Review Board of People's Hospital of Xizang Autonomous Region (ME-TBHP-24-KJ-040) with waiver of informed consent under local regulations. The study adhered to the Strengthening the Reporting of Observational Studies in Epidemiology (STROBE) guidelines. A statistical analysis plan was prespecified prior to formal data modeling, and data analysis was conducted in March 2026.

### 2.2 Setting

This study was conducted at Tibet Autonomous Region People's Hospital in Lhasa, China (altitude 3,650 m), an environment characterized by chronic hypobaric hypoxia. Because the operating rooms do not have an air supply, mechanical ventilation was initiated with 100% oxygen immediately after endotracheal intubation. Lung-protective ventilation strategies were employed, including tidal volumes of 4–8 mL/kg predicted body weight and positive end-expiratory pressure (PEEP) of 0–8 cm H_2_O, adjusted by the attending anesthesiologist based on real-time clinical feedback.

### 2.3 Participants

**Figure 1:**
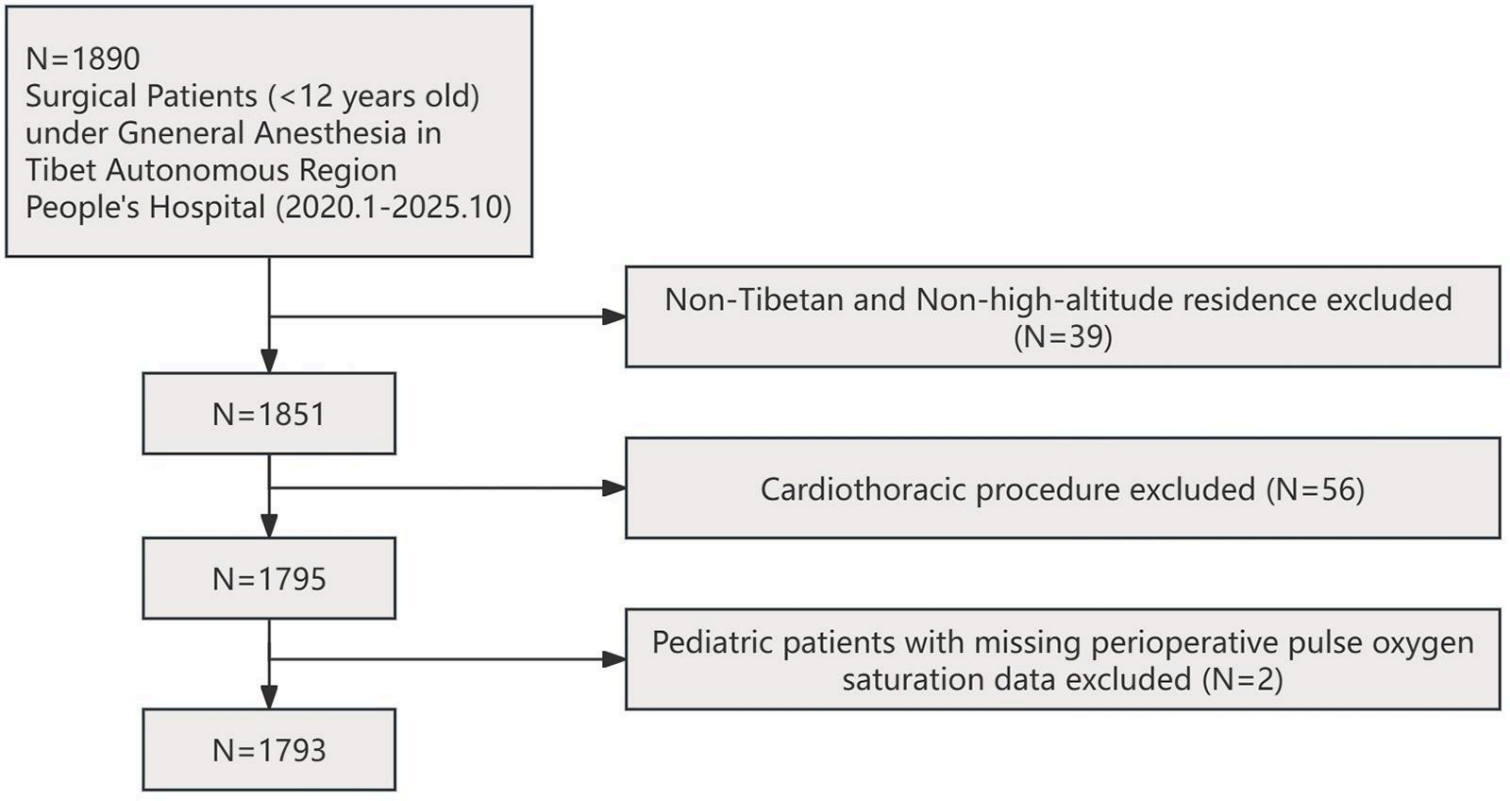
Flowchart for inclusion and exclusion.

Consecutive pediatric patients aged <12 years who underwent elective non-cardiothoracic surgery requiring general anesthesia, endotracheal intubation, and mechanical ventilation between January 2020 and October 2025 were screened (*n* = 1,890) (4). Because physiological adaptation to hypobaric hypoxia varies significantly with the duration of exposure, enrollment was restricted to patients of native Tibetan ethnicity with a documented residential address within the Tibet Autonomous Region, representing lifelong high-altitude acclimatization. After excluding those who did not meet high-altitude residency and ethnic criteria (*n* = 39), cardiothoracic surgery (*n* = 56), and missing oxygen saturation data (*n* = 2), the final cohort included 1,793 patients (Figure 1).

### 2.4 Exposure definitions and data collection

Patient data were systematically extracted from the hospital's Anesthesia Information Management System (AIMS) and cross-referenced with electronic medical records by independent researchers. Extracted covariates included demographic characteristics (age, sex, body weight) (9, 10), preoperative variables [peripheral oxygen saturation (SpO_2_) at rest 1 day before surgery, preoperative hemoglobin levels (defined as the most recent measurement obtained within 30 days before surgery)], surgical details (American Society of Anesthesiologists [ASA] physical status, surgical site), comorbid pulmonary diseases, and the total duration of mechanical ventilation (11, 12).

To accurately assess nutritional and developmental status, absolute body weight was converted into age- and sex-specific weight percentiles. This classification strictly utilized the 2009 standardized growth curves for Chinese children and adolescents to ensure ethnic and regional validity (13). Patients were categorized into severe underweight (<3rd percentile), normal weight (3rd–97th percentile), and overweight (>97th percentile) cohorts (4).

Surgical sites were classified as high-risk or low-risk group based on their distinct potential for intraoperative mechanical impairment of respiratory function (14). Since cardiothoracic surgeries were excluded (*n* = 56), the high-risk group comprised intra-abdominal and head-and-neck surgeries. Intra-abdominal procedures increase intra-abdominal pressure and displace the diaphragm cephalad, reducing intraoperative FRC and pulmonary compliance (15, 16). Head-and-neck surgeries present unique airway management challenges (17, 18). Conversely, the low-risk group included peripheral surgeries, such as orthopedic and superficial surgeries. In our elective cohort, these consisted almost entirely of routine, minor-to-moderate procedures (e.g., closed/open reduction of simple fractures, hardware removal) that do not involve thoracic/abdominal compression or airway manipulation.

Comorbid pulmonary diseases were identified based on preoperative chest radiograph findings (19). Specific radiographic abnormalities constituting a pulmonary comorbidity included exudation, fibrosis, nodules, emphysema, pleural effusion, pneumonia, and interstitial lung changes.

### 2.5 Primary and secondary outcomes

Intraoperative desaturation was defined as SpO_2_ <90% lasting ≥1 min during mechanical ventilation. Secondary outcomes included successful on-table extubation, postoperative intensive care unit (ICU) admission, and total hospital length of stay (LOS).

### 2.6 Statistical analysis

All statistical analyses were performed using R software (R Foundation for Statistical Computing, Vienna, Austria; version 4.2.1). Continuous variables were expressed as mean ± SD or median (IQR) and compared using Student's *t*-test or Mann–Whitney *U*-test. Categorical variables were presented as *n* (%) and evaluated using Chi-square or Fisher's exact test.

To identify independent risk factors for intraoperative desaturation, a multivariable logistic regression model was constructed. Candidate variables were selected *a priori* based on clinical relevance and previous literature. The multivariable model was adjusted for age, sex, body weight percentile (age- and sex-specific, based on the 2009 Chinese growth standards) (13), comorbid pulmonary disease, preoperative SpO₂ at rest, preoperative hemoglobin level, ASA physical status, surgical site, and mechanical ventilation time. Multicollinearity among the included covariates was rigorously assessed using the Variance Inflation Factor (VIF). Results were reported as adjusted odds ratios (ORs) with 95% confidence intervals (CIs).

While the multivariable model screened for general risk factors, we prospectively designated nutritional and developmental status (represented by body weight) as the primary exposure of interest, given its profound physiological implications in pediatric populations (4, 7). To rigorously evaluate the linearity assumption for absolute body weight in the logit scale, a restricted cubic spline (RCS) model with four knots (placed at the 5th, 35th, 65th, and 95th percentiles) was deployed, adjusting for all covariates (20). To identify a potential threshold effect within this non-linear relationship, a two-piecewise logistic regression model was applied. The inflection point was determined by maximizing the model likelihood, and a bootstrap resampling approach was utilized to estimate the 95% CI of the breakpoint. Model performance was evaluated using the C-index (area under the receiver operating characteristic curve) for discrimination and the Hosmer-Lemeshow goodness-of-fit test for calibration. Furthermore, a sensitivity analysis was performed by modifying the weight percentile thresholds to the 10th and 90th percentiles. Subgroup analyses were conducted to evaluate whether the association between severe underweight status (<3rd percentile) and desaturation was consistent across different age strata (e.g., 1–4 years vs. 5–11 years) and sex. All tests were two-sided, and statistical significance was defined as *P* < 0.05.

## 3 Results

### 3.1 Baseline characteristics of the study population

A total of 1,793 pediatric patients who underwent non-cardiothoracic surgery under general anesthesia with mechanical ventilation at high altitude were enrolled. Intraoperative desaturation, defined as at least one episode of SpO_2_ <90% lasting ≥1 min during mechanical ventilation, occurred in 63 patients (3.5%) (Table 1).

**Table 1:** Baseline demographic and clinical characteristics of pediatric patients at high altitude.

Characteristics	Total (*n* = 1,793)	No desaturation (*n* = 1,730)	Desaturation ^a^ (*n* = 63)	*P* value
Age, years	5.9 ± 3.1	5.9 ± 3.0	4.1 ± 2.8	<0.001
Male, *n* (%)	1,060 (59.1)	1,031 (59.6)	29 (46.0)	0.031
Body weight, kg	21.6 ± 8.6	21.8 ± 8.6	16.1 ± 7.2	<0.001
Body weight ^b^			<0.001	
<3rd percentile for age, *n* (%)	87 (4.9)	76 (4.4)	11 (17.5)	
3rd-97th percentile for age, *n* (%)	1,582 (88.4)	1,532 (88.8)	50 (79.4)	
>97th percentile for age, *n* (%)	120 (6.7)	118 (6.8)	2 (3.2)	
SpO_2_ at rest, %	90.0 (89.0, 92.0)	90.0 (89.0, 92.0)	90.0 (89.0, 92.0)	0.984
ASA status			0.736	
I, II, *n* (%)	1,722 (96.0)	1,662 (96.1)	60 (95.2)	
Ⅲ, Ⅳ, Ⅴ, *n* (%)	71 (4.0)	68 (3.9)	3 (4.8)	
Comorbid pulmonary disease, *n* (%)	170 (9.5)	165 (9.5)	5 (7.9)	0.670
Surgical sites			0.289	
Low-risk surgery (Extremity surgery)	361 (20.1)	345 (19.9)	16 (25.4)	
High-risk surgery (Non-extremity surgery)	1,432 (79.9)	1,385 (80.1)	47 (74.6)	
Preoperative hemoglobin, g/dL	13.5 ± 1.8	13.6 ± 1.8	12.6 ± 2.0	<0.001
Mechanical ventilation time, hours	1.7 ± 1.2	1.7 ± 1.2	2.4 ± 1.5	<0.001

Data are presented as mean ± standard deviation, median (interquartile range), or *n* (%). Comparisons between groups used Student's *t*-test or Mann–Whitney *U*-test for continuous variables and *χ*^2^ or Fisher's exact test for categorical variables, as appropriate. Statistical significance was defined as two-sided *P* < 0.05. Surgical groups: “low-risk surgery (Extremity surgery)” includes orthopedic and superficial procedures; “high-risk surgery (Non-extremity surgery)” includes intra-abdominal and head-and-neck procedures. Cardiothoracic surgeries were excluded from the study.

ASA, American Society of Anesthesiologists; SpO_2_, peripheral oxygen saturation.

a Desaturation was defined as at least one episode of SpO_2_ <90% during mechanical ventilation lasting ≥ 1 min.

b Body weight percentiles were determined according to the 2009 standardized growth curves for Chinese children and adolescents.

Patients who experienced desaturation were significantly younger (4.1 ± 2.8 vs. 5.9 ± 3.0 years, *P* < 0.001) and had lower absolute body weight (16.1 ± 7.2 vs. 21.8 ± 8.6 kg, *P* < 0.001) compared to those without desaturation (Table 1). When converted to age- and sex-specific percentiles, severe underweight (weight <3rd percentile for age) was more prevalent in the desaturation group (11/63, 17.5%) than in the non-desaturation group (76/1,730, 4.4%; *P* < 0.001). The desaturation group also presented with significantly lower preoperative hemoglobin levels (12.6 ± 2.0 g/dL vs. 13.6 ± 1.8 g/dL, *P* < 0.001) and endured longer durations of mechanical ventilation (2.4 ± 1.5 h vs. 1.7 ± 1.2 h, *P* < 0.001). No statistically significant differences were observed between the two groups for preoperative SpO_2_ at rest, ASA physical status, surgical procedures, and the presence of comorbid pulmonary diseases.

### 3.2 Risk factors for intraoperative desaturation

In the multivariable model (Table 2), age emerged as a protective factor; each one-year increase in age was associated with a 20% reduction in the odds of desaturation (adjusted OR 0.80, 95% CI: 0.72–0.90; *P* < 0.001). Similarly, higher preoperative hemoglobin levels conferred a protective effect, with a 1 g/dL increment corresponding to a 15% lower risk (adjusted OR 0.85, 95% CI: 0.74–0.98; *P* = 0.021).

**Table 2:** Multivariable logistic regression analysis of risk factors for intraoperative desaturation.

Variable	Crude OR (95%CI)	Crude *P* value	Adjusted OR (95%CI)	Adjusted *P* value
Age (years)	0.81 (0.73–0.89)	<0.001	0.8 (0.72–0.9)	<0.001
Sex (Female)	1.73 (1.04–2.86)	0.033	1.76 (1.01–3.04)	0.045
Body weight for age				
<3rd percentile	4.43 (2.22–8.86)	<0.001	4.57 (2.18–9.58)	<0.001
3rd-97th percentile	Ref.			
>97th percentile	0.52 (0.12–2.16)	0.368	0.37 (0.09–1.56)	0.175
Comorbid pulmonary disease	0.82 (0.32–2.07)	0.671	0.48 (0.18–1.31)	0.154
SpO_2_ at rest (%)	1.02 (0.94–1.12)	0.611	1.02 (0.93–1.12)	0.634
Preoperative hemoglobin levels (g/dL)	0.78 (0.7–0.88)	<0.001	0.85 (0.74–0.98)	0.021
ASA status				
I, II	Ref.			
Ⅲ, Ⅳ, Ⅴ	1.22 (0.37–4)	0.74	0.4 (0.11–1.44)	0.159
Surgical sites				
low-risk surgery (Extremity surgery)	Ref.			
high-risk surgery (Non-extremity surgery)	0.73 (0.41–1.31)	0.291	1.5 (0.79–2.83)	0.213
Mechanical ventilation time (hours)	1.41 (1.21–1.64)	<0.001	1.46 (1.24–1.73)	<0.001

The multivariable model was adjusted for age, sex, body weight percentile, comorbid pulmonary disease, SpO_2_ at rest (%), preoperative hemoglobin levels, ASA status, surgical sites, and mechanical ventilation time. Surgical groups: “low-risk surgery (Extremity surgery)” includes orthopedic and superficial procedures; “high-risk surgery (Non-extremity surgery)” includes intra-abdominal and head-and-neck procedures. Cardiothoracic surgeries were excluded from the study.

OR, odds ratio; CI, confidence interval; Ref., reference; ASA, American Society of Anesthesiologists.

Conversely, extreme low body weight (<3rd percentile) was the strongest independent risk factor, increasing the odds of intraoperative desaturation by 4.57-fold compared to the normal weight cohort (adjusted OR 4.57, 95% CI: 2.18–9.58; *P* < 0.001). Prolonged mechanical ventilation was also a strong predictor; every 1 h extension in mechanical ventilation time escalated the risk by 46% (adjusted OR 1.46, 95% CI: 1.24–1.73; *P* < 0.001). Additionally, female patients independently predicted a higher risk of desaturation relative to male patients (adjusted OR 1.76, 95% CI: 1.01–3.04; *P* = 0.045).

Overall, the multivariable model demonstrated acceptable discrimination (C-index = 0.776, 95% CI: 0.708–0.844) and adequate goodness-of-fit (Hosmer-Lemeshow *P* = 0.199).

### 3.3 Non-linear association between body weight and desaturation

**Figure 2:**
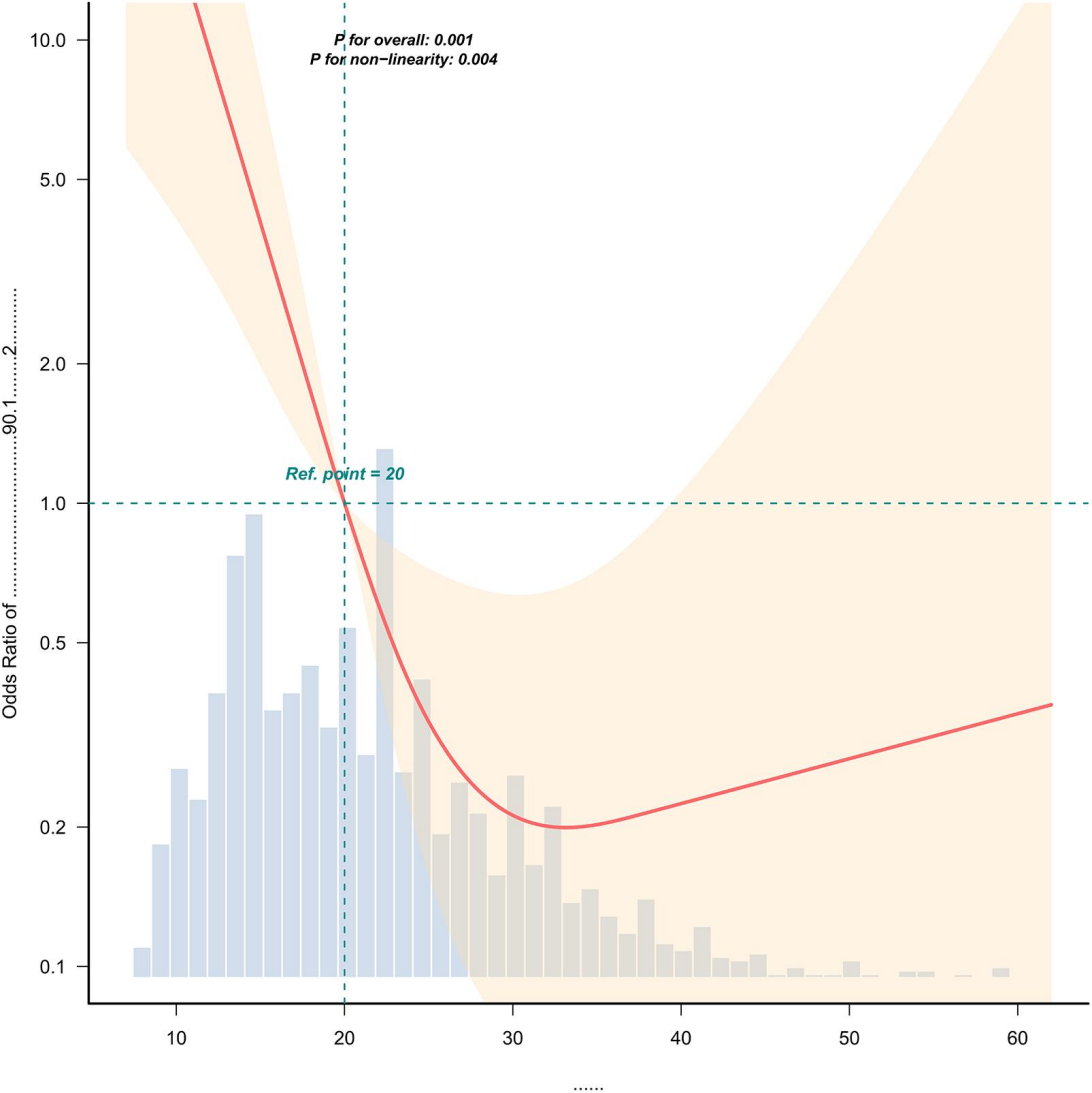
Non-linear association between body weight and intraoperative desaturation. The ﬁt curve shows a non-linear relationship between body weight (kg) and intraoperative desaturation (SpO_2_ <90%) in Tibetan non-cardiothoracic surgical pediatric patients. The solid line indicates the adjusted odds ratio, and the dashed lines indicate the 95% conﬁdence intervals. Adjustment factors included age, sex, body weight, comorbid pulmonary disease, SpO_2_ at rest (%), preoperative hemoglobin levels, ASA status, surgical sites, and mechanical ventilation time.

A significant non-linear association between absolute body weight and the risk of intraoperative desaturation was observed using a restricted cubic spline model (*P* for non-linearity = 0.004; Figure 2). Piecewise logistic regression identified an exploratory statistical inflection point at 26.95 (95% CI: 26.71–27.19) kg (Table 3). This narrow bootstrap confidence interval reflects the mathematical properties of the sharp geometric transition between the two slopes in this specific dataset, rather than establishing a precise threshold with high absolute certainty. Below this threshold, a significant negative association was observed: every 1 kg increase in body weight significantly reduced the adjusted odds of desaturation by 25.6% (adjusted OR 0.744, 95% CI: 0.646–0.857; *P* < 0.001). However, above this threshold, no significant association was observed (adjusted OR 1.055, 95% CI: 0.883–1.260; *P* = 0.554).

**Table 3:** Threshold effect analysis of body weight(kg) on interoperative desaturation in Tibetan Non-cardiothoracic surgical pediatric patients.

Threshold of body weight (95% CI, kg)	OR (95%CI)	*P* value
＜26.95 (26.71, 27.19)	0.744 (0.646–0.857)	<0.001
≥26.95 (26.71, 27.19)	1.055 (0.883–1.26)	0.554
Non-linear Test	–	0.004

Adjusted for age, sex, comorbid pulmonary disease, SpO_2_ at rest (%), preoperative hemoglobin levels, ASA status, surgical sites, and mechanical ventilation time.

OR, odds ratio; CI, confidence interval; ASA, American Society of Anesthesiologists.

### 3.4 Sensitivity analysis

The robustness of our findings was assessed through several sensitivity analyses. First, when the weight percentile threshold was redefined to the 10th and 90th percentiles (Supplementary Table S1), a body weight below the 10th percentile remained a strong independent risk factor (adjusted OR 2.59, 95% CI: 1.43–4.67; *P* = 0.002).

Second, subgroup analysis visualized using a forest plot (Supplementary Figure S1) demonstrated that severe underweight status (<3rd percentile) consistently increased the risk of desaturation across both age and sex subgroups. Interaction testing revealed no significant effect modification by age group (*P* for interaction = 0.213) or sex (*P* for interaction = 0.321).

### 3.5 Postoperative clinical outcomes

Patients in the desaturation group had a significantly lower rate of successful on-table extubation (85.7% vs. 95.4%, *P* = 0.003) and a nearly four-fold higher rate of postoperative ICU admission (12.7% vs. 3.5%, *P* = 0.002) compared to the non-desaturation group (Table 4). The desaturation group experienced a significantly prolonged total hospital LOS (median 13.0 days vs. 9.0 days, *P* < 0.001).

**Table 4:** Comparison of clinical outcomes between patients with and without intraoperative desaturation.

Clinical outcomes	Total (*n* = 1,793)	No desaturation (*n* = 1,730)	Desaturation (*n* = 63)	*P* value
On-table extubation, *n* (%)	1,704 (95.0)	1,650 (95.4)	54 (85.7)	0.003
ICU, *n* (%)	69 (3.8)	61 (3.5)	8 (12.7)	0.002
Postoperative LOS, days	6.0 (3.0, 8.0)	5.0 (3.0, 8.0)	7.0 (5.0, 10.0)	<0.001
LOS, days	9.0 (6.0, 13.0)	9.0 (6.0, 13.0)	13.0 (9.5, 18.5)	<0.001

Data are presented as *n* (%) for categorical outcomes and median (interquartile range) for continuous outcomes. *P* values from *χ*^2^ or Fisher's exact test for categorical variables and Mann–Whitney *U*-test for continuous non-normal variables.

Desaturation was defined as at least one episode of SpO_2_ <90% during mechanical ventilation lasting ≥ 1 min.

Surgical groups: “low-risk surgery (Extremity surgery)” includes orthopedic and superficial procedures; “high-risk surgery (Non-extremity surgery)” includes intra-abdominal and head-and-neck procedures. Cardiothoracic surgeries were excluded from the study (see Figure 1).

On-table extubation: extubation in operating room after procedure.

ICU: postoperative admission to intensive care unit; Postoperative LOS: length of stay after surgery (days); LOS: total hospital length of stay (days).

Model diagnostics: C-index = 0.776 (95% CI: 0.708–0.844); Hosmer-Lemeshow *χ*² = 11.055, *P* = 0.199.

Surgical groups: “low-risk surgery (Extremity surgery)” includes orthopedic and superficial procedures; “high-risk surgery (Non-extremity surgery)” includes intra-abdominal and head-and-neck procedures. Cardiothoracic surgeries were excluded from the study (see Figure 1).

## 4 Discussion

In this large-scale retrospective cohort study of pediatric patients undergoing general anesthesia at 3,650 m altitude, the overall incidence of intraoperative desaturation during mechanical ventilation was 3.5%. Independent risk factors included younger age, severe underweight (<3rd percentile), lower preoperative hemoglobin, prolonged mechanical ventilation, and female sex. Nonlinear dose-response analysis revealed an L-shaped relationship between absolute body weight and desaturation risk, identifying an exploratory developmental inflection point at 26.95 kg. These intraoperative desaturation events showed higher rates of adverse postoperative outcomes in unadjusted between-group comparisons, including delayed extubation, increased ICU admission, and prolonged hospital stay; however, these are exploratory observations and have not been confirmed in confounder-adjusted analyses.

The rationale for using SpO_2_ <90% as the desaturation threshold in this high-altitude cohort warrants specific discussion. The median resting SpO₂ of our cohort was 90% on room air, which is consistent with previous studies showing that the resting SpO₂ of healthy residents at altitudes of 3,800–4,200 m is approximately 90% (21). All patients received 100% FiO_2_ during mechanical ventilation. At 3,650 m, breathing 100% FiO_2_ increases inspired PO_2_ approximately five-fold, which should bring hemoglobin saturation close to its maximal capacity. Therefore an SpO_2_ decline below 90% despite 100% FiO_2_ signals a clinically significant failure of oxygenation rather than a normal altitude-adjusted value (22). Furthermore, the 1 min duration requirement adds specificity by capturing sustained rather than transient events. While Subhi et al. proposed lower, altitude-specific hypoxemia thresholds for awake children based on population percentiles, such as an SpO_2_ below 85% at approximately 3,200 m (23), this standard was derived under fundamentally different physiological conditions (room air, spontaneous breathing, acute illness) and cannot be directly applied to the intraoperative setting. No validated altitude-specific SpO₂ threshold exists for defining intraoperative desaturation under general anesthesia, and this remains an important area for future research.

Anatomically, younger and severely underweight children have smaller airways. This disadvantage is compounded in females due to dysanapsis (24). Females have narrower airways relative to lung volume than males (25). These anatomical features increase airway resistance during mechanical ventilation, predisposing to early airway closure and atelectasis (26, 27). Physiologically, children have a lower ratio of FRC to closing capacity (28). At 3,650 m, the reduced partial PO_2_ further diminishes their respiratory reserve. Moreover, while mild anemia is generally well tolerated at sea level, chronic hypobaric hypoxia triggers compensatory erythrocytosis in high-altitude natives (29, 30). Consequently, a hemoglobin level considered “low” by sea-level standards may reflect inadequate physiological compensation, providing little buffering capacity for oxygenation when anesthesia disrupts normal respiratory mechanics. This accelerates desaturation during surgery.

Compared with previous sea-level cohorts that reported pediatric intraoperative hypoxemia incidences between 3.1% and 12% (2, 3), our observed rate of 3.5% is relatively low. This likely reflects strict inclusion of elective, non-cardiothoracic procedures, the routine use of 100% oxygen for ventilation (necessitated by the lack of an ambient air blending source), and the innate physiological acclimatization of native Tibetan children. Younger age, severe underweight, and prolonged mechanical ventilation are well-established risk factors at sea level as well (4, 11). However, sea-level studies frequently highlight high body weight or obesity as a primary trigger of perioperative desaturation (4, 31). Higher body weight percentiles did not emerge as a significant risk factor in our plateau cohort. This divergence is likely attributable to the intrinsically low prevalence of extreme high body weight in our native Tibetan pediatric population, consistent with previous reports (8, 32). This restricted weight distribution limits the statistical power to detect an association with desaturation, even if a true effect exists. Therefore, the null finding should not be interpreted as evidence that excessive weight poses no risk under hypobaric hypoxia. Further studies in high-altitude populations with greater weight diversity are needed to clarify this relationship.

In unadjusted between-group comparisons, even transient desaturation (SpO_2_ <90% for ≥1 min) was associated with a nearly four-fold higher rate of ICU admission (12.7% vs. 3.5%) and significantly prolonged hospital stay. Although these are process metrics rather than direct measures of tissue-level harm, they represent critical endpoints in resource-limited high-altitude settings, where such adverse events impose a substantial healthcare burden.

The exploratory inflection point of approximately 27 kg provides a data-driven reference for risk stratification in this cohort. This threshold may reflect a critical stage in the maturation of thoracic mechanics, or it may broadly identify the most physiologically vulnerable children. These considerations remain exploratory and require external validation.

This study has several limitations. First, the retrospective, single-center design precludes causal inference and carries a risk of unmeasured confounding. Second, the AIMS did not record several potentially important intraoperative variables, including anesthetic agent type and dose, neuromuscular blocking agent use, specific PEEP levels, dynamic tidal volume adjustments, or intraoperative fluid management. These factors can independently influence intraoperative oxygenation (33–36). However, as a single-center study following standardized institutional protocols where all patients received 100% FiO_2_ via endotracheal intubation with lung-protective ventilation (tidal volumes 4–8 mL/kg based on predicted body weight), the variability in ventilation strategy was limited, reducing the potential impact of unrecorded respiratory parameters. Third, the total number of hypoxemic events (*n* = 63) is modest, raising concerns about the events-per-variable (EPV) ratio. Nevertheless, our primary model maintained an EPV between 5 and 9; simulation studies indicate that logistic regression yields robust estimates within this range without substantial bias (37). Fourth, the postoperative outcomes reported (delayed extubation, ICU admission, prolonged LOS) are process metrics rather than direct measures of patient harm. No data on hypoxic injury-related complications (e.g., hypoxic-ischemic encephalopathy, arrhythmia, cardiopulmonary arrest) or 30-day mortality were available. The absence of such events in this cohort is consistent with its predominantly low-risk profile (96% ASA I–II, elective non-cardiothoracic procedures) and distinguishes our population from critical neonatal cohorts in which mortality associations have been demonstrated (31). Additionally, surgical risk was classified solely by surgical site using a binary system (high-risk vs. low-risk), which does not capture the full spectrum of intraoperative surgical complexity, including estimated blood loss, transfusion requirements, or vasoactive medication use. Although duration of mechanical ventilation was included in the multivariable model as a practical surrogate for overall surgical stress, surgical site was not found to be an independent risk factor (adjusted OR 1.50, *P* = 0.213). To address these limitations, prospective multicenter studies with expanded sample sizes are required to externally validate our findings, incorporate granular surgical risk stratification, and evaluate direct tissue-harm outcomes in higher-risk populations.

In conclusion, our study demonstrates that intraoperative desaturation among pediatric patients at 3,650 m altitude is independently driven by younger age, severe underweight (<3rd percentile), lower preoperative hemoglobin, female sex, and prolonged mechanical ventilation, and is associated with adverse clinical outcomes. Regarding absolute body weight, we identified an exploratory non-linear safety threshold at 26.95 kg, below which respiratory vulnerability escalates dramatically. Future prospective, multicenter studies are essential to validate this threshold and further evaluate whether targeted interventions can effectively mitigate hypoxemic risks.

## Data Availability

The original contributions presented in the study are included in the article/Supplementary Material; further inquiries can be directed to the corresponding authors.
