# Supplementary material for: Intraoperative desaturation in pediatric patients at high altitude: incidence, risk factors, and a non-linear body weight safety threshold
Source: Front Pediatr. 2026 Jun 23;14:1871256. doi: 10.3389/fped.2026.1871256 (PMC13337936; doi:10.3389/fped.2026.1871256)
Supplement: Supplementary file 1 [file Datasheet1.pdf]

Subgroup	Variable	Total	Event (%)	OR (95%CI)	P for interaction
Age (1−4 years)					0.213
	Body weight 3rd−97th	576	36 (6.2)	1(Ref)	
	**<3rd**	**31**	**5 (16.1)**	**3.23 (1.11~9.4)**	
	>97th	74	1 (1.4)	0.19 (0.03~1.46)	
	Trend.test	681	42 (6.2)	0.28 (0.12~0.66)	
Age (5−11 years)					
	Body weight 3rd−97th	1006	14 (1.4)	1(Ref)	
	**<3rd**	**56**	**6 (10.7)**	**7.56 (2.59~22.12)**	
	>97th	46	1 (2.2)	1.53 (0.19~12.21)	
	Trend.test	1108	21 (1.9)	0.18 (0.06~0.54)	
Male					0.321
	Body weight 3rd−97th	932	24 (2.6)	1(Ref)	
	**<3rd**	**56**	**4 (7.1)**	**2.45 (0.79~7.63)**	
	>97th	71	1 (1.4)	0.32 (0.04~2.53)	
	Trend.test	1059	29 (2.7)	0.38 (0.15~0.97)	
Female					
	Body weight 3rd−97th	650	26 (4)	1(Ref)	
	**<3rd**	**31**	**7 (22.6)**	**7.53 (2.73~20.73)**	
	>97th	49	1 (2)	0.37 (0.05~2.92)	
	Trend.test	730	34 (4.7)	0.16 (0.06~0.41)	

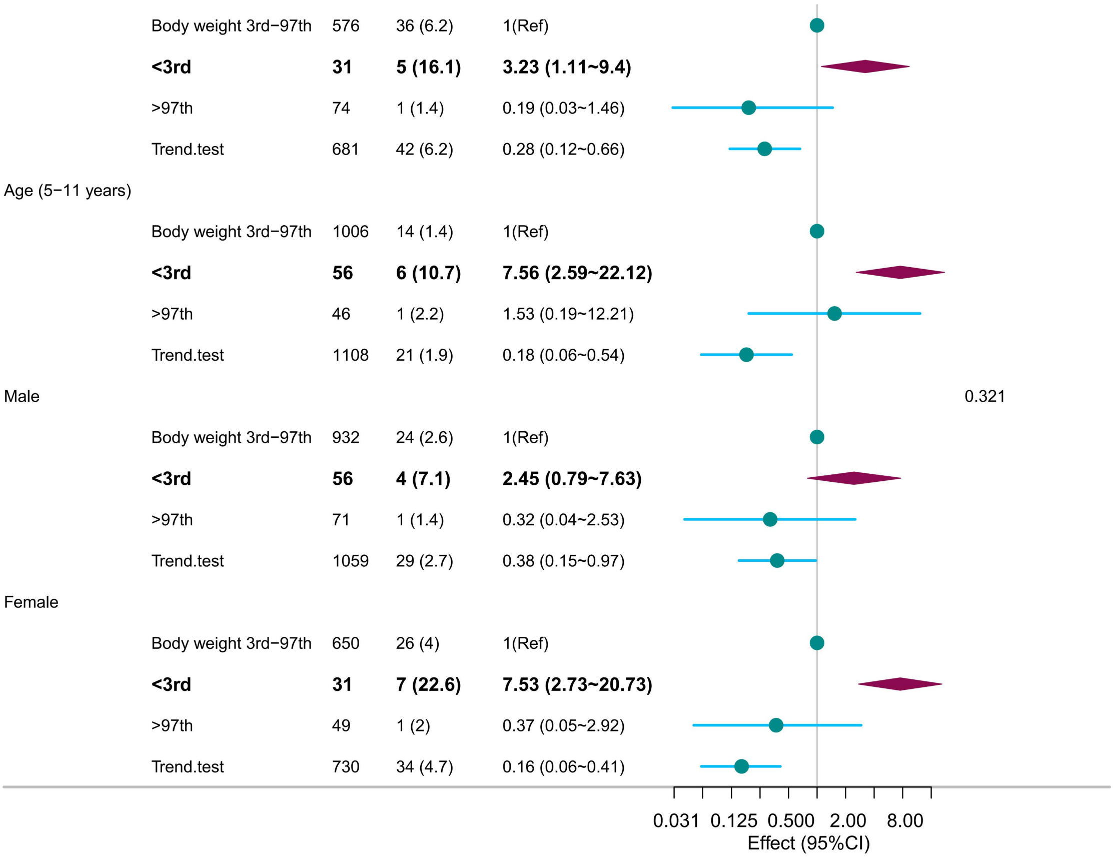

Supplementary Figure 1. Stratified analysis assessing the effect of body weight percentile on intraoperative desaturation.

Effect size of body weight percentile on intraoperative desaturation according to age group and sex. OR, odds ratio; CI, confidence interval.

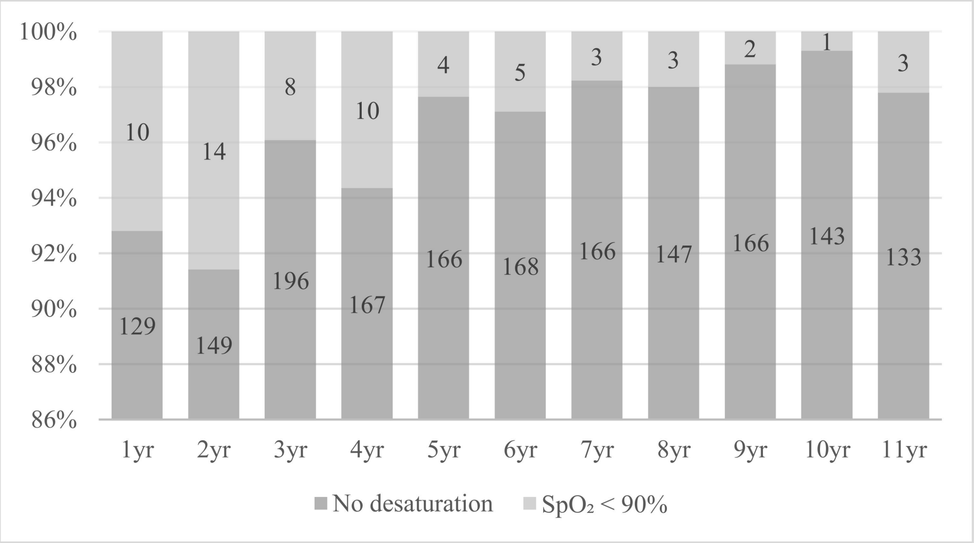

Supplementary figure 2. Incidence of intraoperative desaturation according to age.

Figure legend

Stacked bar chart showing the proportion of patients within each age group who experienced no desaturation or desaturation (SpO_2 < 90% for ≥ 1 minute) during mechanical ventilation. Numbers within bars indicate the count of patients in each category. Age groups are shown on the x- axis; the y- axis denotes the percentage within each age group.

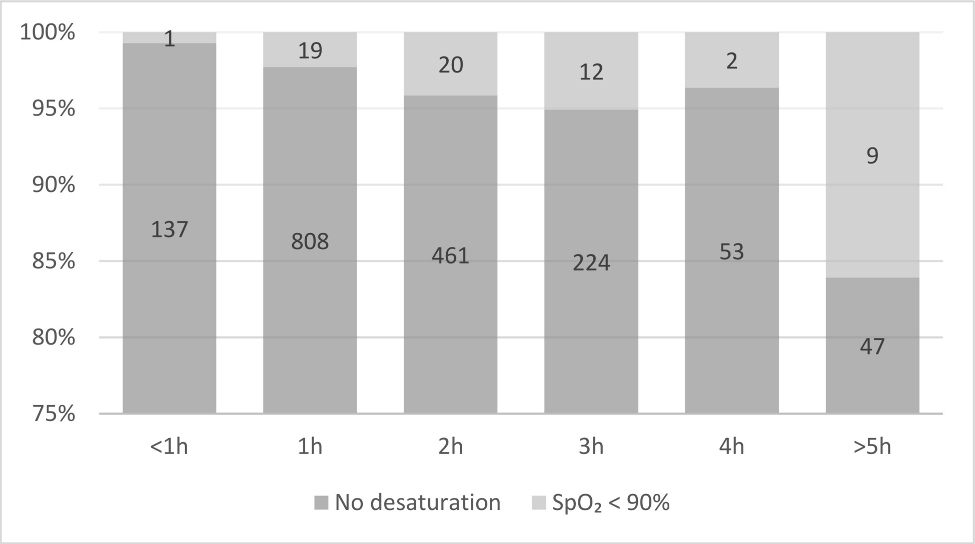

Supplementary figure 3. Incidence of intraoperative desaturation in relation to mechanical ventilation time.

Figure legend

Stacked bar chart showing the proportion of patients within each ventilation duration category who experienced no desaturation or desaturation ($SpO_2 < 90\%$ for ≥ 1 minute) during mechanical ventilation. Numbers within bars indicate the count of patients in each category. Duration categories (<1h to >5h) are shown on the x-axis; the y-axis denotes the percentage within each duration category.
