# Supplementary material for: Intraoperative desaturation in pediatric patients at high altitude: incidence, risk factors, and a non-linear body weight safety threshold
Source: Front Pediatr. 2026 Jun 23;14:1871256. doi: 10.3389/fped.2026.1871256 (PMC13337936; doi:10.3389/fped.2026.1871256)
Supplement: Supplementary file 2 [file Table1.docx]

Supplementary Table 1. Sensitivity analysis using body weight 10^th^ and 90^th^ t percentile thresholds. Univariable and multivariable logistic regression analysis of risk factors for intraoperative desaturation.

| Variable | Crude OR (95%CI) | Crude *P* value | Adjusted OR (95%CI) | Adjusted *P* value |
| --- | --- | --- | --- | --- |
| Age (years) | 0.81 (0.73-0.89) | <0.001 | 0.8 (0.72-0.89) | <0.001 |
| Sex (Female) | 1.73 (1.04-2.86) | 0.033 | 1.57 (0.91-2.72) | 0.106 |
| Body weight for age* |  |  |  |  |
| <10th percentile | 2.61 (1.48-4.6) | 0.001 | 2.59 (1.43-4.67) | 0.002 |
| 10th-90th percentile | Ref. |  |  |  |
| >90th percentile | 0.7 (0.27-1.79) | 0.457 | 0.53 (0.2-1.39) | 0.195 |
| Comorbid pulmonary disease | 0.82 (0.32-2.07) | 0.671 | 0.55 (0.21-1.45) | 0.229 |
| SpO_2_ at rest (%) | 1.02 (0.94-1.12) | 0.611 | 1.02 (0.93-1.13) | 0.641 |
| Preoperative hemoglobin levels (g/dL) | 0.78 (0.7-0.88) | <0.001 | 0.85 (0.74-0.98) | 0.026 |
| ASA status |  |  |  |  |
| I, II | Ref. |  |  |  |
| Ⅲ, Ⅳ, Ⅴ | 1.22 (0.37-4) | 0.74 | 0.42 (0.11-1.51) | 0.182 |
| Surgical sites |  |  |  |  |
| Head and neck surgery | Ref. |  |  |  |
| Peripheral surgery | 1.17 (0.63-2.2) | 0.616 | 0.63 (0.32-1.25) | 0.187 |
| Abdominal surgery | 0.71 (0.39-1.29) | 0.26 | 0.85 (0.46-1.6) | 0.62 |
| Mechanical ventilation time (hours) | 1.41 (1.21-1.64) | <0.001 | 1.46 (1.23-1.73) | <0.001 |

The multivariable model was adjusted for age, sex, body weight percentile, comorbid pulmonary disease, SpO_2_ at rest (%), preoperative hemoglobin levels, ASA status, surgical sites, and mechanical ventilation time.

Abbreviations: OR, odds ratio; CI, confidence interval; Ref., reference.

*: Age-specific body weight percentiles were determined according to the 2009 standardized growth curves for Chinese children and adolescents.
